# Anemia before reimplantation surgery: An overlooked modifiable risk factor of septic revision knee arthroplasty failure

**DOI:** 10.1051/sicotj/2020046

**Published:** 2020-12-11

**Authors:** Faustine Bredeche, Isabelle Gounot, Vincent Belgaïd, Caroline Macabeo, Kaissar Rouhana, Frederic Aubrun, Tristan Ferry, Elvire Servien, Sebastien Lustig, Mikhail Dziadzko

**Affiliations:** 1 Département d’Anesthésie-Réanimation, Hôpital de la Croix-Rousse, Hospices Civils de Lyon, Université Claude Bernard 69004 Lyon France; 2 Orthopaedics Surgery and Sports Medicine Department, FIFA Medical Center of Excellence, Croix-Rousse Hospital, Lyon University Hospital 103 Grande rue de la Croix Rousse 69004 Lyon France; 3 Centre Interrégional de Référence Pour la Prise en Charge des Infections Ostéo-Articulaires Complexes (CRIOAc Lyon), Hôpital de la Croix-Rousse 93 Grande Rue de la Croix-Rousse 69004 Lyon France; 4 Service des Maladies Infectieuses et Tropicales, Hospices Civils de Lyon, Hôpital de la Croix-Rousse 93 Grande Rue de la Croix-Rousse 69004 Lyon France; 5 EA 7425 HESPER, Université Claude Bernard Lyon 1 69003 Lyon France; 6 Univ Lyon, Claude Bernard Lyon 1 University, IFSTTAR, LBMC UMR_T9406 69622 Lyon France

**Keywords:** anemia, surgical site infection, revision arthroplasty, patient blood management, perioperative medicine

## Abstract

*Introduction*: Preoperative anemia in patients undergoing a two-stage septic revision arthroplasty may be a factor of reinfection, even in the presence of aggressive antimicrobial therapy. Patient Blood Management (PBM) in such patients is challenging. We evaluate the impact of anemia existing before re-implantation on a failure rate after two-stage septic total knee arthroplasty (rTKA), and explore feasibility of a PBM strategy implementation in these patients. *Materials and methods*: A retrospective study of patients from January 2010 to January 2015 in a French regional referral center was performed. Patients undergoing a two-stage rTKA for infection after successful primary TKA were identified and followed up to 31.12.2018. The primary outcome (failure) was defined as surgical site infection after re-implantation requiring new surgery. The secondary outcomes were time to failure, the time between explantation/reimplantation, transfusion rate during the second stage. Preoperative anemia was defined as Hb level < 12 g/L before the re-implantation. *Results*: 69 patients were identified; 17 (24%) developed reinfection of rTKA in 105 [11.4–156] days. In these patients pre-implantation anemia was more frequent (*n* = 13(76.5%) in failed vs. *n* = 21(40%) in non-failed, *p* = 0.0110). During the explantation stage, there were no significant group differences in age, sex, comorbidity, type of spacer and antimicrobial therapy, iron supplementation, or transfusion rate. The median time between explantation/reimplantation surgery was 51 [43–71.5] days, indifferent between the two groups. Intraoperative transfusion during reimplantation was required in 12 (17%) patients, more frequent in failed patients. None of the patients had contraindications for the PBM strategy except the cell-saver use. *Conclusion*: In two-stage septic rTKA preoperative anemia was almost two times more frequent and associated with an elevated rate of septic failure. The time-frame between explantation and-re-implantation is sufficient to implement a PBM strategy for all anemic patients. Before-after studies would be of interest to determine the best PBM strategy to prevent anemia-associated septic failure in such a condition.

## Introduction

The management of surgical site infection (SSI) following arthroplasty may be challenging, often needs multiple surgeries including revision, aggressive antimicrobial therapy, and extended hospital stay. It is associated with a higher incidence of morbidity and mortality and increased cost of care [1]. Re-infections after septic revision surgery are more frequent comparing to SSI after primary arthroplasty, and therefore are a real concern for surgeons [2]. The revision arthroplasty rate is increasing for the past two decades, with sepsis as the main cause of revision [3, 4] and the decrease for mechanical reasons [5].

Patients who underwent septic revision arthroplasty are more likely to have complications including deep venous thrombosis, surgical site infection, and death. The risk of re-infection after septic (mostly two-stage) revision TKA is considerably higher than infection after primary TKA [6]. Multiple predictors associated with secondary surgical site infections following septic revision were reported such as age, comorbidities, surgery time, and transfusion [7]. In large retrospective cohorts, the reported rate of failed septic revision (reinfection) is 7.6% in one-stage revision and 8.8% for two-stage revision [8].

Preoperative anemia is a well-established independent and modifiable factor of postoperative morbidity after primary arthroplasty [9, 10]. However, the role of preoperative anemia as a risk factor of recurrent SSI after septic revisions, especially two-stage revisions with aggressive antimicrobial therapy, is less evident.

Patient Blood Management (PBM) programs are widely used to address preoperative anemia in patients undergoing primary THA/TKA. The transversal approach of the PBM includes perioperative iron supplementation, the use of antifibrinolytics, lower transfusion triggers, aggressive hydration protocols, regional anesthesia, minimally or anatomical surgical access/incision, and the use of bipolar sealer. The combination of all these strategies results in improved outcomes [11]. However, in two-stage septic revisions, the PBM is often limited by minimization of blood loss, and it’s not enough for patients already suffering from anemia [12]. Commonly applied extended antimicrobial therapy may also be an aggravating co-factor of anemia due to the intestinal microbiota impairment. No single strategy is recommended to be superior over another in reducing the need for blood transfusion in these patients.

We conducted a retrospective cohort study to evaluate the impact of anemia existing before the second stage on a failure in two-stage septic TKA arthroplasty and to explore the feasibility of a PBM strategy implementation in these patients. Our hypothesis was a negative role of anemia on a septic failure after two-stage revision TKA and a time-frame between explantation and re-implantation surgery large enough to benefit from the PBM program.

## Materials and methods

We conducted a retrospective cohort study of patients from the Lyon CRIOAC database [13] from January 2010 to January 2015 in a French regional referral center.

Patients who underwent a two-stage revision knee surgery for an SSI after the successful primary (first rang) TKA were identified and followed up to 31.12.2018. A failure (primary outcome) was defined as a necessity for the second surgery for the deep infection after the re-implantation. The secondary outcomes were time to failure, the time between explantation and reimplantation surgery, transfusion rate during the second stage.

Preoperative anemia was defined as a Hb level below 12 g/L before the second-stage surgery (re-implantation).

We analyzed the process of access to the re-implantation surgery: time from the TKA sepsis diagnosis to pre-anesthesia consultation, time from pre-anesthesia consultation to the explantation surgery, the time between explantation and reimplantation, the time between second pre-anesthesia consultation and reimplantation surgery. The following patient’s data were collected: demographic data, comorbidities for the Charlson Index calculation, American Society of Anesthesiologists (ASA) physical status before the first surgery, presence and type of spacer, type of antimicrobial therapy between explantation and re-implantation surgery, hemoglobin level up to three days before the explantation and re-implantation surgery; surgery length, blood loss, and transfusion rate during both stages. All data were collected from the institutional electronic health record system.

### Statistical analysis

A comparison between patients with failure after revision and no failure was performed using Wilcoxon or Fisher’s exact test, as appropriate. A two-tailed *p*-value < 0.05 was used to define statistical significance. Statistical analyses were performed using JMP 11 (SAS, Cary, NC) software.

### Ethics consideration

Our work is a part of the implemented prospective observational cohort study Bone Joint Infection Lyon (NCT02817711) received the approval of the French South-East ethics committee with the reference number CAL2011-021. All patients included in this study received informed consent of their medical data use. In accordance with French legislation, a written patient’s agreement was not required for any part of the study.

## Results

We identified 69 patients who underwent a two-stage rTKA after the first successful prosthetic knee surgery, with a median age of 68 [62.5–76] years. Seventeen patients (24%) developed reinfection of rTKA in 105 [11.4–156] days. There were no significant differences in age, sex, and comorbidity (Table 1).

**Table 1:** General characteristics of patients included in the observation.

	Total	Failed	Not failed	
	*n* = 69	*n* = 17 (24.6%)	*n* = 52 (75.4%)	
Sex	38 (55%) F	10 (26%)	28 (74%)	*p* = 0.7844
	31 (45%) M	7 (22.6%)	24 (77%)	
Age	68 [62.5–76]	72 [64.5–77.5]	67.5 [61.25–76]	*p* = 0.4240
BMI	29 [25.5–33]	29 [27–32.5]	30 [25–34]	*p* = 0.9784
ASA	I – 1 (1.4%)	I – 0 (0%)	I – 1 (2%)	*p* = 0.0883
	II – 35 (50.7%)	II – 9 (53%)	II – 26 (50%)	
	III – 31 (45%)	III – 6 (35%)	III – 25 (48%)	
	IV – 2 (2.9%)	IV – 2 (12%)	IV – 0 (0%)	
Charlson Index	4 [3–5]	4 [3–5.5]	4 [2.25–5]	*p* = 0.5561
Time to event or follow up, weeks	239 [165–329]	105 [11.4–156]	269 [217–343]	*p* < 0.001

Median [interquartile range]; BMI – body mass index; ASA – American Society of Anesthesiologists physical status.

There was a preponderance of streptococci in patients with failed rTKA (35% vs. 2% in non-failed), while in non-failed patients there were more methicillin-sensible *Staphylococcus* strains (27%). In about 25% of patients from both groups, a microbial agent was not identified (Table 2). The total number of antimicrobial agents used in patients during the time between the two stages was non-significantly different in both groups (Table 3).

**Table 2:** Principal pathogens found in the infected joint after explantation.

	Failed		Non failed	
	*n* = 17		*n* = 52	
Streptococcus	6	35%	1	2%
*Sterile*	4	24%	15	29%
MSSE	2	12%	4	8%
*Propionibacterium*	1	6%	3	6%
MRSA	1	6%	3	6%
*E. coli*	1	6%	0	0%
*Mycobacterium*	1	6%	0	0%
MSSE + *Klebsiella*	1	6%	0	0%
MSSA	0	0%	9	17%
MSSE	0	0%	6	12%
BGP	0	0%	1	2%
*Corynebacterium*	0	0%	1	2%
*E. faecalis* + MSSA	0	0%	1	2%
*Klebsiella*	0	0%	1	2%
*Propionibacterium + P. aeruginosa*	0	0%	1	2%
MSSA + *E. faecalis*	0	0%	1	2%
MSSA + *Propionibacterium*	0	0%	1	2%
MSSA + *Propionibacterium + E. cloacae*	0	0%	1	2%
MSSA + *P. aeruginosa*	0	0%	1	2%
MSSA + *Streptococcus*	0	0%	1	2%
MSSE + *Propionibacterium*	0	0%	1	2%

BGP – Bacilli Gram-positive; MSSA – methicillin-sensitive *S. aureus*; MRSA – methicillin-resistant *S. aureus*; MSSE – methicillin-sensitive *S. epidermidis*.

**Table 3:** Number of different antimicrobial agents used in both groups between explantation-implantation surgeries.

N ATB (single agent)	Failed		Non-failed	
	*n* = 17		*n* = 52	
1	1	6%	1	2%
2	5	29%	6	12%
3	1	6%	13	25%
4	7	41%	18	35%
5	2	12%	10	19%
6	1	6%	3	6%
7	0	0%	1	2%

ATB – a single antibiotic.

Before the first stage of surgery (explantation), anemia was more frequent in 13/17 patients (76.5%) who developed reinfection of TKA whereas anemia was present in 23/52 patients (46%), who have been not failed *p* = 0.0475. There were no significant differences in iron therapy (*p* = 0.7782) or transfusion rate (*p* = 1) in the two groups (Table 4).

**Table 4:** Explantation surgery data.

	Total	Failed	Not failed	
	*n* = 69	*n* = 17 (24.6%)	*n* = 52 (75.4%)	
Time from pre-anesthesia evaluation to explantation, days	14 [6–25.5]	14 [5.5–46.5]	14 [6–26.75]	*p* = 0.1370
Spacer w/ATB	53 (77%)	15 (88%)	38 (73%)	*p* = 0.3222
Hemoglobin level	119 [109–134]	119 [110–122]	120 [108–134]	*p* = 0.5306
Anemia	31 (46%)	13 (76.5%)	23 (46%)	*p* = 0.0475
Transfusion	18 (26%)	4 (23%)	18 (26%)	*p* = 1
IV Iron supplementation	40 (58%)	9 (53%)	31 (60%)	*p* = 0.7782

Median [interquartile range]; ATB – antibiotic; IV – intravenous.

Before the second stage of surgery (re-implantation) anemia was more frequent in failed patients – 13 (76%) versus 21 (40%) in non-failed, with a significant difference (OR 3.82 [1.09–13.33, *p* = 0.0475). The median time between explantation and reimplantation surgery was 51 [43–71.5] days, 47 [43-54] in the failed group, and 52.5 [43–82.5] in the successful group, not significantly different (*p* = 0.1832). None of the patients had contraindications for the PBM strategy except cell-saver use. Twelve patients (17%) required intraoperative transfusion during reimplantation. Blood transfusion was more frequent in failed patients (35% vs. 11% accordingly; *p* = 0.0585) (Table 5).

**Table 5:** Reimplantation surgery data.

	Total	Failed	Not failed	
	*n* = 69	*n* = 17 (24.6%)	*n* = 52 (75.4%)	
Time from pre-anesthesia evaluation to implantation, days	14 [2–25.5]	6 [1–20]	16 [2–27.5]	*p* = 0.0980
Time between two stages, days	51 [43–71.5]	47 [43–54]	52.5[43–82.5]	*p* = 0.1832
Surgery time, min	130 [111.5–143]	132 [122.5–153.5]	123 [108.5–140]	*p* = 0.2074
Ciment w/ATB	53 (77%)	15 (88%)	38 (73%)	*p* = 0.3222
Hemoglobin level	120 [111.5–128.5]	112 [104.5–121.5]	121.5 [115–131]	*p* = 0.0110
Anemia	34 (49%)	13 (76%)	21 (40%)	*p* = 0.0125
Blood loss, mL	420 [335–650]	600 [325–1035]	415 [332.5–597.5]	*p* = 0.2251
Transfusion	12 (17%)	6 (35%)	6 (11%)	*p* = 0.0585
IV Iron supplementation	46 (67%)	13 (77%)	33 (63%)	*p* = 0.3872

Median [interquartile range]; ATB – antibiotic; IV – intravenous.

## Discussion

The main finding of our study is the confirmation of a negative role of anemia on a septic failure after two-stage revision TKA in a small cohort of patients. All patients had a time-frame between explantation and re-implantation surgery large enough to benefit from the PBM program.

Preoperative anemia and perioperative transfusion result in increased morbidity and mortality both in elective general and orthopedic surgery. In a large retrospective study [10] in patients with septic revision, preoperative anemia was associated with a two-fold increase of the risk of total complications (OR 2.16 95% CI [1, 83–2.56] *p* < 0.001). Although the pre-existing anemia in patients with primary arthroplasties and revision arthroplasties for mechanical failure is addressed through the different implemented PBM, this is not a generalized practice in septic revision patients.

Anemia is defined as a decrease below a defined threshold at the blood count, of the hemoglobin of a subject. Normal hemoglobin varies with age and sex in adults. Although the diagnosis of anemia is positive at < 13 g/dL for males and < 12 g/dL for females [14], we have purposely chosen the unique hemoglobin threshold at 12 g/dl. Anemia was already present in more than half of patients with SSI. Irons supplementation alone between the first and second stages was not efficient in these patients, has not led to anemia correction or reduction in transfusion rate, which is in accordance with the most recent study [15].

**Figure 1:**
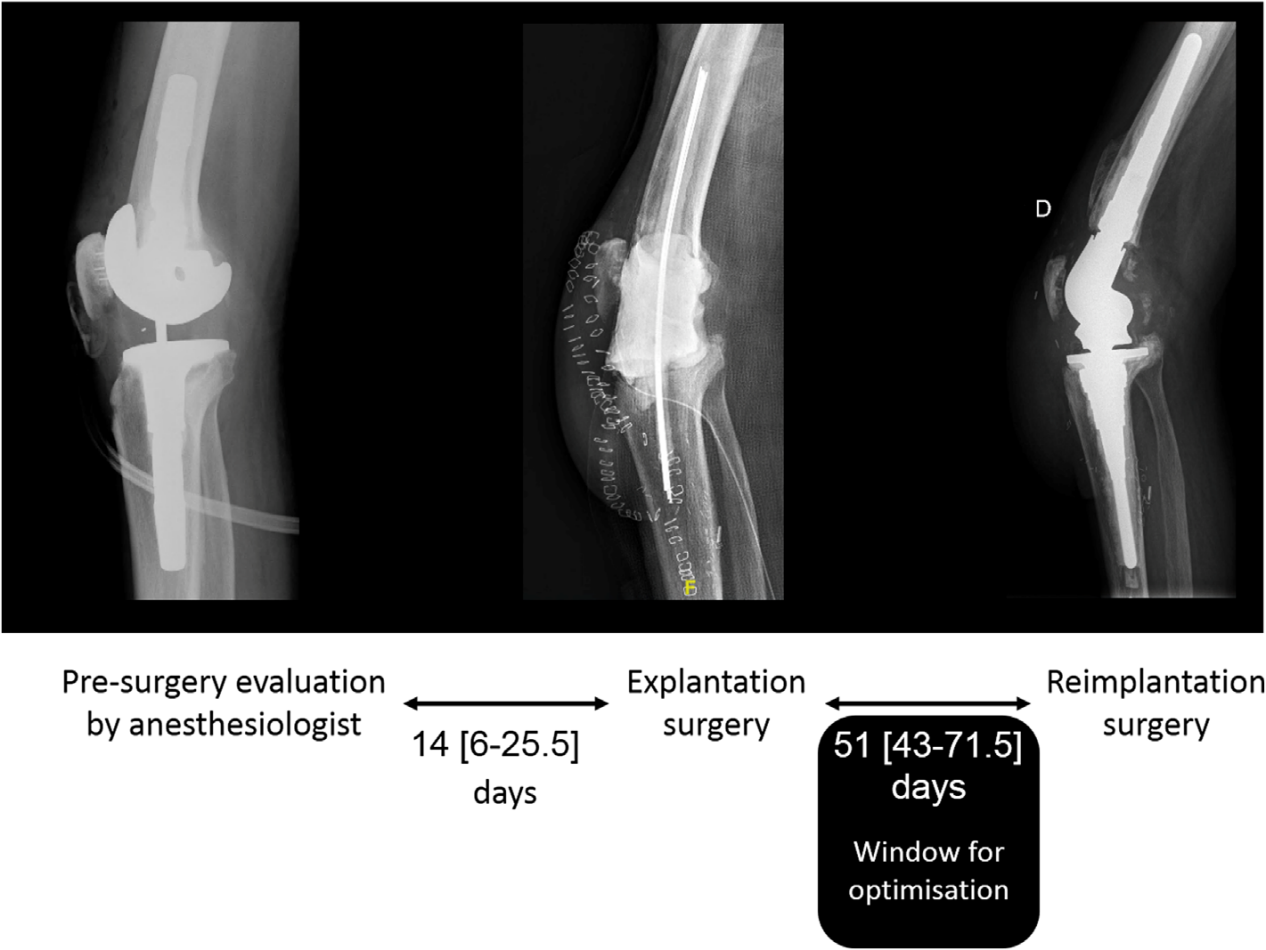
Analysing the large time-frame influencing window to optimize the patient for the surgery.

All patients had a pre-anesthesia evaluation, which is mandatory before each scheduled intervention. In our observation, the time from pre-anesthesia evaluation to surgery was about 2 weeks both for the explantation and re-implantation surgery. The delay between the explantation and reimplantation surgery was important (7 weeks). This large time-frame is an opportunity window to optimize the patient for the surgery (Figure 1). None of our patients had benefited from erythropoietin (EPO) administration associated with iron supplementation in the period between explantation and implantation. The reason for such omission is unknown. In our opinion, this practice may reflect the overlooking of the anemia factor in the context of urgent and septic surgery, in the presence of aggressive antimicrobial therapy.

The concept of Patient Blood Management [16] is recommended [17–19] during the three stages of surgical care: pre, per, and postoperatively. According to the guidelines [17], in scheduled surgery preoperative hemoglobin levels should be assessed approximately 4 weeks prior to surgery. All deficiencies have to be corrected by iron and vitamin supplementation, and/or EPO. Therefore, a minimum of three weeks before the procedure is required. The use of EPO is validated by the National Drug Safety Agency [20] in moderately anemic patients before scheduled orthopedic surgery. Two regimens are available: 4 administrations of EPO (600 UI/kg) with one subcutaneous injection per week, starting 21 days before the procedure, or 10 daily administrations (300 UI/kg) 10 days prior to the surgery. An oral or IV martial treatment must be associated.

The delay between the explantation and reimplantation stages observed in our patients allows applying this strategy largely. The anesthesiologist may play a coordination role as a perioperative practitioner. However, no studies evaluating the efficiency of the EPO therapy for anaemia correction in two-stage septic revision arthroplasty are available yet.

Regarding the microbial strains found, no meaningful comparison between the two groups as possible. However, the duration and antibiotic regimens used in the two groups were comparable. The antimicrobial therapy was managed by Infectious Diseases specialists and microbiologists.

The main limitation of the study is its retrospective nature with a small number of patients analyzed. No power analysis was made for the primary end-point. However, the homogeneity of the cohort with no follow-up loss reinforces our conclusions, which are consistent with current data from the literature.

The reinfection after revision TKA is a rare but serious event. Such patients are often followed at specialized referring centres. Our study covers 5 years of the follow-up, and exhaustively studies cases of septic relapse after the first explantation-reimplantation of TKA from the Rhone Alpes region, France, through the Lyon CRIOAC. These observations have led to the development of an institutional PBM protocol specifically designed for patients with revision orthopaedic surgery.

## Conclusion

In patients undergoing the rTKA in the context of two-stage septic revision preoperative anemia before the reimplantation was observed in half of cases, and was associated with an elevated rate of septic failure. The time-frame between explantation and-re-implantation surgeries is sufficient to implement a PBM strategy for all anemic patients. Before-after studies would be of interest to determine the best PBM strategy to prevent anemia-associated septic failure in patients undergoing two-stage septic revision of TKA.

## Conflict of interest

The authors declare that they have no conflict of interest.
